# Effect of a digital health intervention on outpatients with heart failure: a randomized, controlled trial

**DOI:** 10.1093/ehjdh/ztaf063

**Published:** 2025-06-10

**Authors:** David O Arnar, Bartosz Dobies, Elias F Gudmundsson, Heida B Bragadottir, Gudbjorg Jona Gudlaugsdottir, Audur Ketilsdottir, Hallveig Broddadottir, Brynja Laxdal, Thordis Jona Hrafnkelsdottir, Inga J Ingimarsdottir, Bylgja Kaernested, Axel F Sigurdsson, Ari Isberg, Svala Sigurdardottir, Tryggvi Thorgeirsson, Saemundur J Oddsson

**Affiliations:** Cardiovascular Services, Landspitali—The National University Hospital of Iceland, Hringbraut Reykjavik 101, Iceland; School of Health Sciences, University of Iceland, Reykjavik 101, Iceland; Department of Life Sciences, Sidekick Health, Vallakor 4, Kopavogur 200, Iceland; Doctoral School of Medical and Health Sciences, Jagiellonian University Medical College, Krakow, Poland; Department of Life Sciences, Sidekick Health, Vallakor 4, Kopavogur 200, Iceland; Department of Life Sciences, Sidekick Health, Vallakor 4, Kopavogur 200, Iceland; Cardiovascular Services, Landspitali—The National University Hospital of Iceland, Hringbraut Reykjavik 101, Iceland; Cardiovascular Services, Landspitali—The National University Hospital of Iceland, Hringbraut Reykjavik 101, Iceland; Cardiovascular Services, Landspitali—The National University Hospital of Iceland, Hringbraut Reykjavik 101, Iceland; Cardiovascular Services, Landspitali—The National University Hospital of Iceland, Hringbraut Reykjavik 101, Iceland; Cardiovascular Services, Landspitali—The National University Hospital of Iceland, Hringbraut Reykjavik 101, Iceland; School of Health Sciences, University of Iceland, Reykjavik 101, Iceland; Cardiovascular Services, Landspitali—The National University Hospital of Iceland, Hringbraut Reykjavik 101, Iceland; School of Health Sciences, University of Iceland, Reykjavik 101, Iceland; Cardiovascular Services, Landspitali—The National University Hospital of Iceland, Hringbraut Reykjavik 101, Iceland; Outpatient Clinic, Reykjavik Heart Center, Kopavogur 200, Iceland; Department of Life Sciences, Sidekick Health, Vallakor 4, Kopavogur 200, Iceland; School of Health Sciences, University of Iceland, Reykjavik 101, Iceland; Department of Life Sciences, Sidekick Health, Vallakor 4, Kopavogur 200, Iceland; Center for Mindfulness, Clinical Medicine, Aarhus University, Aarhus, Denmark; Department of Life Sciences, Sidekick Health, Vallakor 4, Kopavogur 200, Iceland; Department of Life Sciences, Sidekick Health, Vallakor 4, Kopavogur 200, Iceland

**Keywords:** Clinical outcome, Digital health programme, Heart failure, Lifestyle-change, Remote monitoring, Self-care

## Abstract

**Aims:**

Heart failure (HF) is associated with high mortality and reduced quality of life (QoL). Interventions encouraging a healthy lifestyle and self-care can reduce morbidity and HF-related hospitalizations. We conducted a randomized controlled trial (RCT) to assess the impact of a digital health programme on QoL and clinical outcomes of patients. The programme included remote patient monitoring (RPM), self-care, HF education, and empowered positive lifestyle changes.

**Methods and results:**

Patients (*n* = 175) received standard-of-care (SoC) at a HF outpatient clinic (control, *n* = 89) or SoC plus a digital health programme (intervention, *n* = 86) for 6 months, followed by a 6-month maintenance period. Compliance with RPM was 93% at 6 months. No significant between-group difference was found in the primary endpoint (health-related QoL), except in an exploratory subgroup of New York Heart Association class III patients, where the intervention group had a significantly smaller QoL decline (*P* = 0.023). For secondary endpoints, the intervention group had significantly greater improvements in self-care at 6 months (*P* < 0.001) and 12 months (*P* = 0.003), and in disease-specific knowledge at 12 months (*P* = 0.001). Several exploratory endpoints favoured the intervention, with significant improvements in triglycerides (*P* = 0.012), HbA1c (*P* = 0.014), and fasting glucose (*P* = 0.010). The TG/HDL cholesterol ratio and TG/glucose index improved significantly at both 6 and 12 months in between-group comparisons.

**Conclusion:**

Although the digital programme did not improve health-related QoL, it led to benefits in other important outcomes such as self-care, disease-specific knowledge, and several key metabolic parameters.

## Graphical Abstract

**Graphical Abstract:**
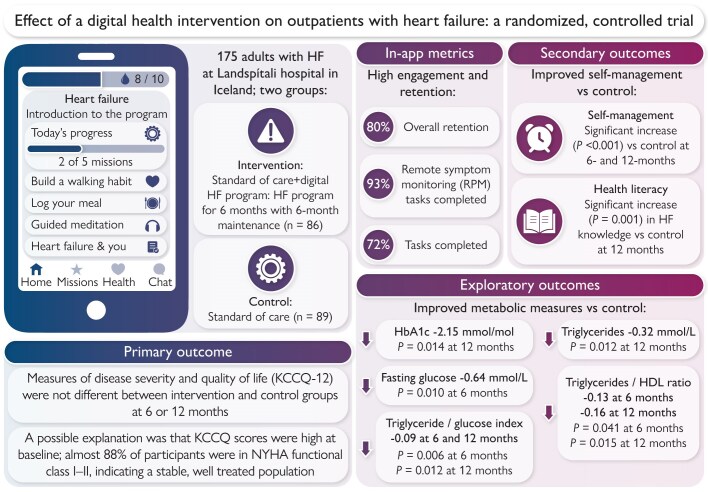

## Introduction

Heart failure (HF) results in a markedly reduced quality of life (QoL) and a high 5-year mortality rate (>50%).^1^ HF also has a high risk of hospitalization, which is further increased by the frequent presence of multiple comorbidities.^2^ The prevalence of HF is about 1–2% in adults, ranging from 1% in adults <55 years old to >10% in adults ≥70 years old.^1^ Around 57–72 million people worldwide have been diagnosed with HF,^3^ and these numbers are increasing due to population growth and the ageing of populations.^4^ Together, these issues lead to high costs and a big burden on healthcare.

The risk of developing HF greatly decreases with a healthy lifestyle, as large prospective studies have shown.^5–7^ In these studies, a healthy lifestyle was defined based on scores for lifestyle factors, such as smoking status, alcohol consumption, quality of diet, physical activity, and body weight. In individuals with established HF, interventions that encouraged a healthy lifestyle reduced mortality as well as the frequency and duration of HF-related hospitalizations.^8–10^ Knowledge and support are essential for the development of successful self-care in HF. Self-care includes factors such as adherence to treatment, certain health maintenance behaviours, and individual monitoring of signs and symptoms of the disease.^11^ Meta-analyses have shown that interventions focused on improving self-care in HF can improve QoL and reduce the risk of hospitalization and mortality.^12–16^ In addition, a systematic review of 29 clinical trials found that multi-disciplinary management strategies, which include support to improve patients’ self-care activities and specialized follow-up care, reduce the risk of HF hospitalizations, overall hospitalizations and mortality, and save costs.^12^ Another systematic review concluded that multi-disciplinary interventions may reduce the risk of readmission for HF or for any cause.^17^ Multi-disciplinary support of self-care is, therefore, one of the recommended interventions in the 2022 guidelines for the management of HF that was published jointly by the American College of Cardiology, the American Heart Association, and the Heart Failure Society of America.^11^

Multi-disciplinary management strategies with specialized follow-up and a focus on improving patient self-care are complex and require increased patient support and healthcare resources. Non-invasive digital technologies, such as telemedicine, can provide patient support, education, remote patient monitoring (RPM), and medical care in a cost-effective, real-time manner. Recently, the American Heart Association published a scientific statement encouraging telehealth use in the treatment of HF patients.^18^ The randomized controlled trial (RCT), TIM-HF2, is an excellent example of a holistic approach that combines RPM of multiple physiological measures and symptoms of HF patients with patient education, assessment of concomitant medications, and delivery of actionable feedback.^19^ Three subgroup analyses of the trial’s participants found a reduction of days lost due to cardiovascular hospitalizations, a reduction in all-cause and cardiovascular mortality, and an improvement in QoL.^20–22^ A limitation of that trial was that the home telemonitoring system, which included a digital tablet and four measuring devices, had to be installed in patients homes,^19^ which increases costs and may limit the number of potential users. Given that approximately two-thirds of the world’s population owns a smartphone,^23^ digital interventions delivered via a smartphone application (app) will facilitate the delivery of specialized follow-up and support that is readily accessible at almost any time and location to many people at a low cost.

We previously developed a digital health programme, delivered through a smartphone app for HF patients, SK-141, that was very well received by patients and showed promising clinical results in a small feasibility study.^24^ The current study aimed to evaluate whether a 6-month intervention with this digital health programme, followed by a 6-month maintenance period, improves the QoL and key clinical outcomes of HF patients compared with controls. Additionally, the goal was to determine how receptive patients were to this rather novel approach.

## Methods

### Trial design and ethics

This study was a single-centre, RCT with patients receiving guidelines-recommended standard-of-care (SoC) therapy at the HF outpatient clinic at Landspitali University Hospital in Reykjavik. Patients were recruited from 26 November 2021 to 14 October 2022, and assessed for eligibility by research nurses or physicians. All patients signed an informed consent form before any study-specific activities. Data were collected at baseline, 3, 6, and 12 months. Due to the nature of the intervention, it was not possible to blind patients, nurses, or physicians. The analyses were conducted by a statistician blinded to the group allocation that was only revealed after the completion of the statistical report. The study protocol was approved by the Icelandic Scientific Ethics Committee (application number: VSN-21-154), the Scientific Research Committee for Health Research of Landspitali University Hospital and was pre-registered in the ClinicalTrials.gov database (NCT05193344).

### Eligibility criteria

Patients were included if they were ≥18 years old, had been diagnosed with HF (New York Heart Association [NYHA] class I-IV), and had started their medical treatment at least 1 month before enrolling in the study. Patients were required to have the ability to understand written and verbal instructions in Icelandic, to own and be able to operate a smartphone and to be willing and capable of complying with study procedures and attending the scheduled visits. Patients were excluded if they had reversible causes of HF, HF due to severe aortic valve stenosis, planned cardiac transplant surgery, kidney failure with eGFR <15 mL/min or planned dialysis in the next 6 months, another serious illness (e.g. cancer), cognitive impairment, or an active drug/alcohol abuse problem.

### Baseline characteristics and randomization

After obtaining informed consent, baseline data were collected, which included demographic information such as gender, age, education status, and body mass index (BMI), as well as clinical information such as NYHA functional classification, left ventricular ejection fraction (EF) (measured by echocardiogram), smoking status, blood pressure, laboratory test results, comorbidities, and medications used. Additionally, the participants completed a 6-minute walking test (6MWT) and various questionnaires. Patients were randomized at a 1:1 ratio using electronic Case Report Form (eCRF) software (Greenlight Guru Clinical, formerly known as SMART-TRIAL) by the research nurses at the outpatient clinic. The randomization was stratified by gender and NYHA class (I-II vs. III-IV). The research nurses entered baseline information into the eCRF system.

### Intervention

**Figure 1:**
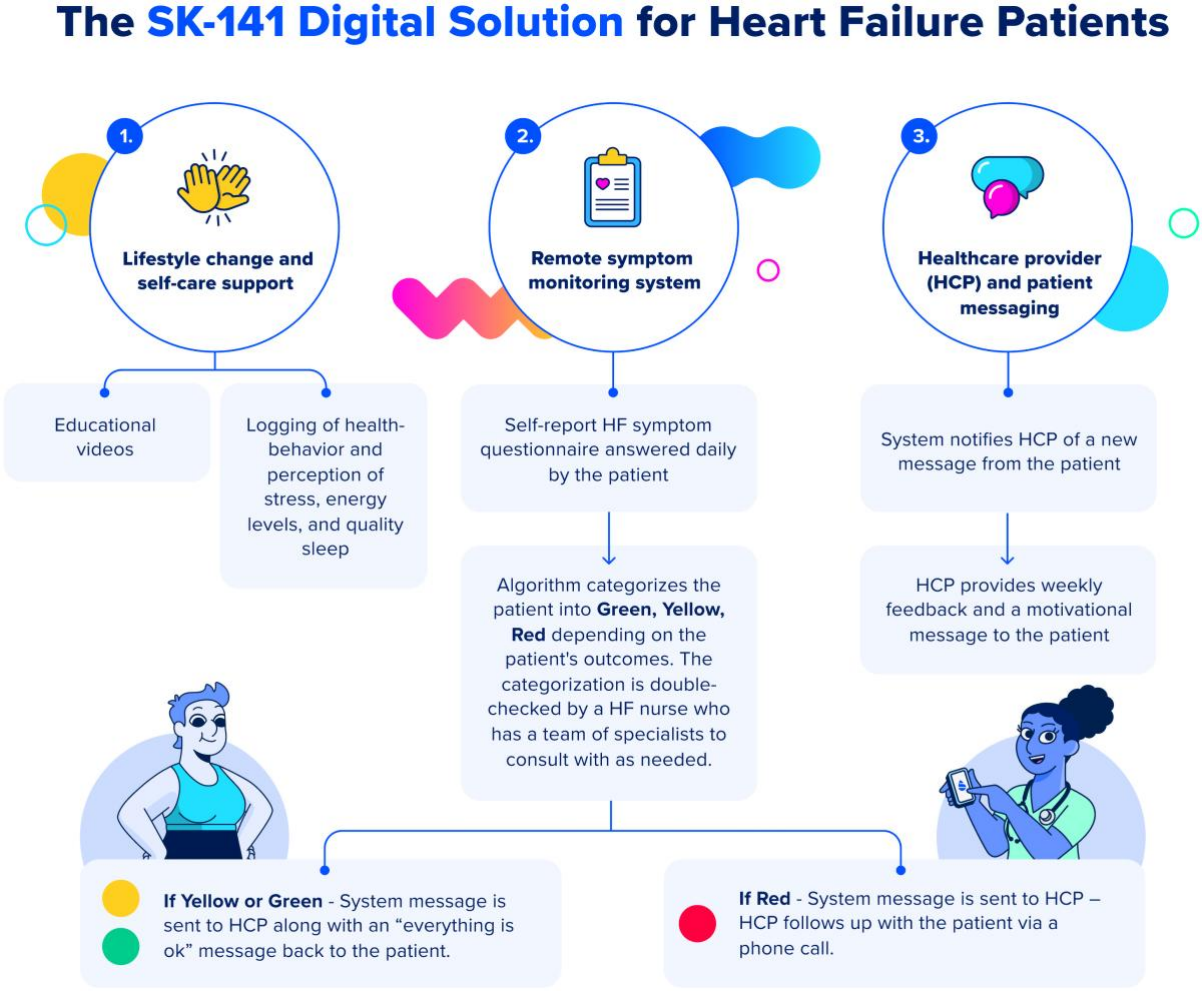
Schematic overview of the digital solution.

All patients received SoC treatment at the outpatient clinic. At the baseline visit, patients in the intervention group additionally gained access to a digital health programme that was delivered via a smartphone application (app). Participants received instructions on how to download and install the app. The digital health programme, delivered remotely via the Sidekick Health app over 48 weeks, had three key components (*Figure 1*): RPM, educational content (including lifestyle and self-care support) and a healthcare provider-patient messaging feature.

For the first 12 weeks, participants reported symptoms daily, which were then monitored by nurses who provided follow-up based on a scoring system. Nurses reviewed RPM data three times daily during working hours, excluding weekends and holidays. Patients completed five daily multiple-choice questions on HF symptoms: breathlessness, fatigue, leg oedema, chest pain, and dizziness. An algorithm classified responses into a three-tiered colour code (green, yellow, and red), guiding clinical response. Green indicated stability, yellow required increased monitoring, and red triggered immediate contact and further action. RPM frequency was gradually reduced to once weekly by weeks 24–52, though daily reporting remained optional.

The patients had the option of using their own monitoring devices for weight and vital signs and to manually enter measurements. Step counts were automatically transferred via smartphone.

The educational programme, spanning weeks 1–24, covered topics like nutrition, medication adherence, sleep, physical exercise, stress management, mental health promotion, and knowledge about HF, with participants completing daily tasks, such as watching educational videos, and reading content cards. From weeks 25–48, they received a recap, with access to earlier content for review. The approach focused on increasing patients’ self-efficacy and autonomy.

### Outcome measures

All patient-reported outcomes were completed independently by the patients and reviewed for completeness by research nurses. The primary outcome was the change in health-related QoL measured by the Kansas City Cardiomyopathy 12-item Questionnaire (KCCQ-12) from baseline to the conclusion of the 6-month intervention. The KCCQ-12 is a validated tool for assessing to what extent patients are limited by their medical condition to perform specific activities over the past 2 weeks. It contains four subdomains: physical limitation, symptom frequency, QoL, and social limitations.^25^

The secondary outcomes of the study involved changes in scores on several other questionnaires: the 9-item European Heart Failure Self-care Behaviour scale (EHFScB9),^26^ the 6-item disease-specific knowledge questionnaire for HF,^27^ the 8-item Morisky Medication Adherence Scale (MMAS-8),^28–30^ and the 21-item Depression, Anxiety, and Stress (DASS-21).^31^

Other secondary outcomes were changes in the number of five important metabolic syndrome conditions (defined as the presence of elevated triglycerides, high fasting glucose, low high-density lipoprotein (HDL) cholesterol, increased waist circumference, and elevated systolic blood pressure), NYHA functional class, N-terminal pro-b-type natriuretic peptide (NT-proBNP), hospital utilization due to cardiovascular disorders (CVD) (number of visits to the emergency room [ER] and admissions to a hospital ward, length of stay), score on the 6MWT^32^ and changes from baseline to 6-month endpoint in EF and left atrial diameter measured by echocardiogram. HF was sub-classified as HF with a reduced EF (HFrEF, EF ≤40%) and HF with a preserved EF (HFpEF, EF ≥50%).^11^

The triglyceride-to-HDL cholesterol ratio and the triglyceride–glucose index were calculated. The former is an indicator of metabolic health and a predictor of future cardiovascular events,^33^ and the latter is an indicator of insulin resistance and a risk marker for future cardiovascular events.^34^ Exploratory outcomes included the EuroQol 5 dimensions 5 levels (EQ-5D-5L, rescaled from 0–1 to 0–100 range) to assess QoL that also includes the visual analogue scale (EQ-VAS) to determine overall health on the day of the interview,^35^ changes from baseline to the 6- or 12-months endpoint in the proportion of participants with an adequate score on the EHFScB9 scale, individual metabolic parameters (including triglycerides (mmol/L), fasting glucose (mmol/L), HbA1c (mmol/mol), HDL cholesterol (mmol/L), left-arm systolic blood pressure (mmHg), waist circumference (cm), the triglyceride-to-HDL cholesterol ratio, and the triglyceride–glucose index) and changes in the proportion of participants with a normal fasting glucose range. An adequate score on the EHFScB9 scale is defined as a score ≥70.25. Normal fasting blood glucose concentrations are defined as fasting glucose between 70 mg/dL (3.9 mmol/L) and 100 mg/dL (5.6 mmol/L).^36^ All models were adjusted for baseline values of the respective outcome to reduce potential bias due to pre-existing differences between groups and to ensure that estimated effects reflected true changes over time, rather than initial imbalances at baseline, thereby improving the validity of group comparisons.

### Sample size calculation

In the previous feasibility study involving the digital health programme for HF, effect sizes of *d* = 0.29 for the total score and *d* = 0.45 for the symptom subscale were observed using the KCCQ-12 questionnaire.^23^ For the sample size calculation in this study, we assumed a medium effect size (*d* = 0.45) based on the larger effects observed in symptom-related outcomes, as these were expected to be more responsive to the intervention due to its RPM component. This assumption is supported by prior research, including the HOM-HEMP RCT, which evaluated a nurse-led, home-based self-management digital programme for patients with HF. While effect sizes were not explicitly reported, the authors based their sample size on a medium effect size, with 80% power and a 5% significance level, accounting for a 25% attrition rate in a final three-arm sample of 213 participants.^37^ Using the R function *samplesize mixed* from the *sjstats* package, and accounting for an anticipated 10% dropout rate, it was calculated that both treatment groups (intervention and control) would require 87 participants. This resulted in a total sample size of 174 participants, achieving a power of 80% at an α-level of 0.05, with Cohen's *d* set at 0.45 for the effect size.

### Statistical analysis

Statistical analyses were performed using R (v. 4.3.2), with α = 0.05 for two-sided tests. Baseline characteristics were summarized using descriptive statistics: continuous variables as means with standard deviations (SD) or medians with first and third quartiles (Q1, Q3), and categorical variables as frequencies and percentages.

*Post hoc* estimates from the fitted models were used to examine between-group contrasts of interest and to report group-specific estimates using R package *emmeans*. The *post hoc* analysis was stratified by time, with each time point analysed separately to assess both shorter- and longer-term effects. The *P*-values of the interaction between time and group were computed to assess the presence of differential group changes over time and inform the interpretation of *post hoc* tests. No formal correction for multiple comparisons was applied, therefore, given the exploratory nature of analyses of multiple secondary and exploratory endpoints, the results should be interpreted with caution.

Primary and secondary outcomes were assessed through modified Intention-to-Treat (mITT) and Per-Protocol (PP) analyses (for PP analysis results see Supplementary material online). The mITT population included all participants who had at least one visit, while PP was limited to those who completed all visits and adhered to the intervention for at least 75% of the programme.

A mixed-model repeated measures (MMRM) approach, adjusted for baseline, age, sex, NYHA class, and education, was used for longitudinal outcomes, incorporating time-group interactions. Participant-specific random intercepts and an unstructured covariance matrix were applied.

Linear mixed models were used for continuous endpoints, and generalized linear mixed models assessed count, ordinal and binary outcomes. Specifically, negative binomial mixed models were applied for count endpoint (metabolic syndrome conditions), logistic mixed models for binary outcomes (EHFScB9 adequate score, normal fasting glucose range), and cumulative link mixed model for ordinal outcome (NYHA class). In cumulative link mixed models, we estimated the cumulative probability of being in lower NYHA class (I or II) vs. higher (III or IV). Group estimates reflect these cumulative probabilities, and contrasts represent between-group differences in these probabilities. For binary outcomes analysed using mixed-effects logistic regression models, risk ratios (RRs) were derived instead of odds ratios (ORs), as the outcomes were common and ORs would overestimate the effect size. Continuous endpoints with only one post-baseline measurement were analysed using ANCOVA adjusted for the same covariates as repeated-measurements models.

To account for data skewness and extreme values, NT-proBNP values were log-transformed due to strong right-skewness, and estimated marginal means, along with standard errors and confidence intervals, were back-transformed (via exponentiation) to the original scale, representing geometric means. Robust methods were applied in selected models to address influential observations: the 6MWT distance was analysed using robust ANCOVA (via the *rlm* function from the *MASS* package), and the triglyceride/HDL cholesterol ratio was analysed using a robust linear mixed model (via the *rlmer* function from the *robustlmm* package).

Negative binomial models evaluated cumulative hospital utilization events (ER visits, hospital admissions, length of stay). Models were adjusted for the cumulative number of respective events over the 12 months prior to the intervention start and for baseline covariates including age, gender, NYHA class, education, number of comorbidities and number of reported medications. Cumulative length of stay was analysed in a model adjusted only for the cumulative number of hospital days during the 12 months prior to the programme, age, and gender due to convergence issues when including additional covariates.

Missing values in MMRM were handled using maximum likelihood estimation, and for outcomes with two measurements, complete cases were analysed with ANCOVA models. Details about the modelling approaches are also mentioned below the results tables. All results were presented with 95% confidence intervals (95% CI).

## Results

### Participants

**Figure 2:**
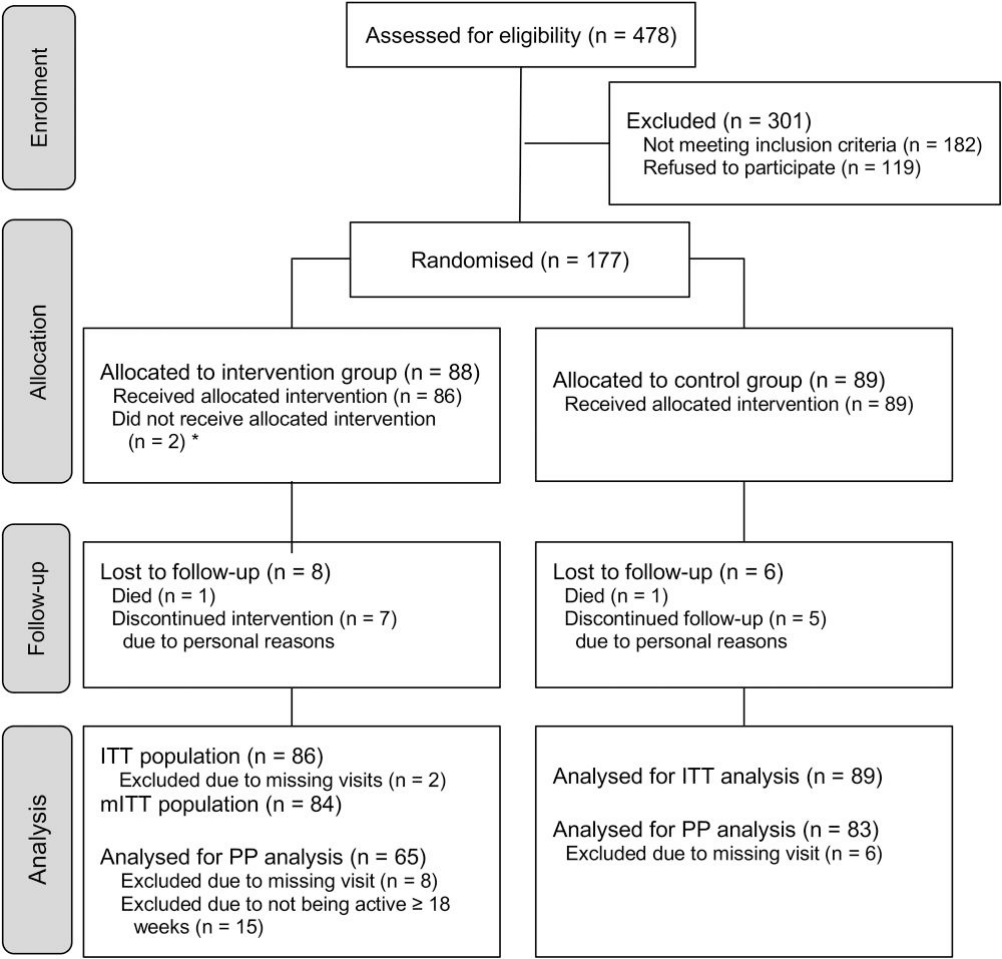
Study participant CONSORT flowchart (main intervention phase). *Due to not being interested (discontinued, did not receive app). CONSORT, consolidated standards of reporting trials; ITT, intention-to-treat; mITT, modified intention-to-treat; *n*, number; PP, per-protocol.

A total of 478 patients were assessed for eligibility; of these, 182 did not meet the inclusion criteria, and 119 were not willing to participate. Thus, 177 participants were enrolled and randomized to either the intervention group (*n* = 88) or the control group (*n* = 89). The mITT analysis included 175 participants as two participants did not complete the baseline assessments. The PP analysis included 148 participants, as 29 participants were excluded due to missing visits or not using the smartphone app after baseline (in the intervention group) (see Supplementary material online). A flow diagram of participant enrolment, allocation, and analysis is depicted in *Figure 2*.

The mean age of participants was 65.5 years (SD 12.2), and 68% were male. Their mean BMI was 31.1 kg/m^2^, and 87.4% were in NYHA functional class I-II. Most participants were not actively employed (57.7%) and were married or cohabitating (68.6%). The intervention and control groups had comparable demographic and clinical characteristics at baseline except for education, which was higher in the control group (education was therefore added to the adjustment factors in later analyses). All demographic and clinical characteristics at baseline are presented in *Table 1*. Baseline values for the outcome characteristics were comparable overall (*Table 2*).

**Table 1:** Demographic and clinical characteristics at baseline

Variable	All patients	Intervention	Control
	(*n* = 175)	(*n* = 86)	(*n* = 89)
Gender, *n* (%)			
Female	56 (32.0)	29 (33.7)	27 (30.3)
Male	119 (68.0)	57 (66.3)	62 (69.7)
Age in years, mean (SD)	65.49 (12.16)	64.71 (13.15)	66.24 (11.13)
BMI in kg/m^2^, mean (SD)	31.06 (5.70)	31.50 (6.29)	30.65 (5.06)
NYHA classification, *n* (%)			
Stage 1	63 (36.0)	29 (33.7)	34 (38.2)
Stage 2	90 (51.4)	46 (53.5)	44 (49.4)
Stage 3	22 (12.6)	11 (12.8)	11 (12.4)
Smoking status, *n* (%)			
Current	17 (9.7)	7 (8.1)	10 (11.2)
Former (>1 month)	104 (59.4)	51 (59.3)	53 (59.6)
Never smoked	54 (30.9)	28 (32.6)	26 (29.2)
Blood pressure in mmHg, mean (SD)			
Systolic	129.77 (22.35)	130.97 (23.45)	128.62 (21.31)
Diastolic	73.64 (13.25)	74.64 (13.14)	72.67 (13.35)
Education status, *n* (%)			
University degree	44 (25.1)	17 (19.8)	27 (30.3)
Trades/vocational school or equivalent	62 (35.4)	29 (33.7)	33 (37.1)
Secondary/matriculate	25 (14.3)	16 (18.6)	9 (10.1)
Primary or less	44 (25.1)	24 (27.9)	20 (22.5)
Working status, *n* (%)			
Not on the labour market	101 (57.7)	47 (54.7)	54 (60.7)
Full-time	46 (26.3)	23 (26.7)	23 (25.8)
Part-time	28 (16.0)	16 (18.6)	12 (13.5)
Family status, *n* (%)			
Married	95 (54.3)	46 (53.5)	49 (55.1)
In cohabitation	25 (14.3)	11 (12.8)	14 (15.7)
Divorced/separated	24 (13.7)	13 (15.1)	11 (12.4)
Single	18 (10.3)	10 (11.6)	8 (9.0)
Widowed	13 (7.4)	6 (7.0)	7 (7.9)
Comorbidities, *n* (%)			
Atrial fibrillation	93 (53.1)	42 (48.8)	51 (57.3)
Hypertension	92 (52.6)	42 (48.8)	50 (56.2)
Hypercholesterolemia	60 (34.3)	27 (31.4)	33 (37.1)
Coronary artery disease	59 (33.7)	28 (32.6)	31 (34.8)
Sleep apnea	51 (29.1)	25 (29.1)	26 (29.2)
Obesity	40 (22.9)	19 (22.1)	21 (23.6)
Diabetes (type 1 or 2)	39 (22.3)	19 (22.1)	20 (22.5)
Peripheral artery disease	12 (6.9)	6 (7.0)	6 (6.7)
Depression	30 (17.1)	17 (19.8)	13 (14.6)

BMI, body mass index; *n*, number; NYHA, New York Heart Association; SD, standard deviation.

**Table 2:** Baseline values of the outcomes

Variable	Intervention (*n* = 86)	Control (*n* = 89)
KCCQ-12, mean (SD)		
Overall summary score [0–100]	64.82 (21.98)	62.40 (26.01)
Clinical summary score [0–100]	66.85 (22.73)	66.39 (24.70)
EHFScB9 [0–100], mean (SD)	53.68 (22.60)	56.86 (23.50)
Adequate (≥70)	23 (27%)	29 (33%)
6-question HF-specific questionnaire [6–30], mean (SD)	22.15 (5.35)	22.16 (4.93)
6MWT in m, mean (SD)	436.47 (114.17)	439.67 (109.65)
(Missing)	1	0
MMAS-8 [0–8], mean (SD)	7.34 (0.85)	7.22 (1.20)
High [8] *n* (%)	46 (53%)	48 (54%)
Moderate [6–7.9], *n* (%)	33 (38%)	29 (33%)
Low [0–5.9], *n* (%)	7 (8.1%)	12 (13%)
DASS-21, mean (SD)		
Total score [0–126]	20.60 (16.66)	19.96 (18.62)
Anxiety sub-score [0–42]	4.91 (3.90)	4.85 (5.08)
Depression sub-score [0–42]	7.86 (8.72)	7.71 (8.45)
Stress sub-score [0–42]	7.84 (5.90)	7.39 (7.56)
Ejection fraction (%), mean (SD)	43.59 (12.91)	45.13 (13.70)
Preserved, *n* (%)	30 (35%)	36 (41%)
Mildly reduced, *n* (%)	17 (19%)	22 (24%)
Reduced, *n* (%)	39 (46%)	31 (35%)
(Missing)	1	1
Left atrium size in cm, mean (SD)	4.53 (0.82)	4.45 (0.70)
(Missing)	2	6
NT-proBNP in pg/mL, median (Q1, Q3)	422 (171, 1345)	357 (128, 1036)
log(NT-proBNP), mean (SD)	6.10 (1.46)	5.83 (1.38)
Metabolic syndrome conditions, mean (SD)		
Score [0–5]	2.58 (1.23)	2.60 (1.08)
(Missing)	0	1
Triglycerides in mmol/L, mean (SD)	1.66 (1.37)	1.58 (0.91)
Fasting glucose in mmol/L, mean (SD)	6.77 (2.52)	6.20 (1.30)
Normal range (3.9–5.6 mmol/L), *n* (%)	28 (33%)	36 (40%)
Above normal range, *n* (%)	58 (67%)	53 (60%)
HbA1c in mmol/mol, mean (SD)	43.79 (12.19)	41.84 (8.84)
HDL cholesterol in mmol/L, mean (SD)	1.38 (0.32)	1.38 (0.44)
Systolic blood pressure in mmHg, mean (SD)	130.97 (23.45)	128.62 (21.31)
Waist circumference in cm, mean (SD)	113.04 (13.99)	111.61 (14.35)
(Missing)	0	1
Triglyceride/HDL cholesterol ratio, mean (SD)	1.37 (1.63)	1.33 (1.02)
Triglyceride–glucose index, mean (SD)	0.24 (0.13)	0.25 (0.14)
EQ-5D-5L [0–100], mean (SD)	80.47 (15.16)	80.09 (16.87)
EQ-VAS [0–100], mean (SD)	69.26 (19.12)	69.56 (21.19)

The numbers in square brackets next to a variable name represent the score range.

DASS-21, 21-item depression, anxiety and stress scale; EHFScB9, nine-item European heart failure self-care behaviour scale; EQ-VAS, EQ visual analogue scale; EQ-5D-5L, 5-level EQ-5D version; HbA1c, haemoglobin A1c; HDL, high-density lipoprotein; Q1, first quartile; Q3, third quartile; KCCQ-12, Kansas City Cardiomyopathy Questionnaire-12; MMAS-8, eight-item Morisky Medication Adherence Scale; *n*, number; NT-proBNP, N-terminal pro-hormone of brain natriuretic peptide; SD, standard deviation; 6MWT, six-minute walking test.

### Retention and engagement

The median compliance rate with RPM, which included reporting of all requested symptom parameters, was 93% at 6 months, while in-app overall programme retention reached 80% at 12 months. In the intervention group, 77% of patients (65/84) were active for at least 84 days, corresponding to engaging with the programme approximately every other day. Over 6 months, users completed 72% of the assigned tasks. After the maintenance phase of an additional 6 months, compliance with the RPM questionnaire (once a week) remained high, with a median compliance rate of 89% over the whole study period. The median task compliance with the educational part (videos and content cards) of the programme was 72% at 6 months.

### Health-related QoL

The primary outcome was the change in health-related QoL measured by the KCCQ-12, from baseline to the conclusion of the 6-month intervention. The changes in overall summary scores between baseline and the 6-month point were not different between the intervention and control groups and thus the primary endpoint was not met (*Table 3*).

**Table 3:** Results of estimated changes from baseline and between-group differences in primary and secondary endpoints for the mITT sample

Variable		Estimated change from baseline (95% CI)		Difference, vs. control (95% CI)	*P* ^a^	Interaction *P*^a^
		Intervention	Control			
Primary endpoint^b^						
KCCQ-12 [0–100]	Month 3	−2.35 (−6.08, 1.37)	−1.59 (−5.37, 2.20)	−0.77 (−5.53, 4.00)	0.752	0.703
	Month 6	−0.35 (−4.10, 3.40)	1.53 (−2.29, 5.35)	−1.88 (−6.70, 2.94)	0.443	
	Month 12	0.40 (−3.41, 4.21)	1.55 (−2.30, 5.39)	−1.15 (−6.04, 3.74)	0.644	
Secondary endpoints^b^						
EHFScB9 [0–100]	Month 6	3.82 (0.46, 7.19)	−4.24 (−7.63, −0.84)	8.06 (3.71, 12.4)	**<0**.**001**	**<0**.**001**
	Month 12	5.95 (2.51, 9.39)	−0.88 (−4.32, 2.55)	6.84 (2.39, 11.3)	**0**.**003**	
6-question HF questionnaire [6–30]	Month 3	0.13 (−0.71, 0.96)	−0.17 (−1.01, 0.66)	0.30 (−0.77, 1.37)	0.582	**0**.**003**
	Month 6	0.77 (−0.07, 1.61)	0.32 (−0.53, 1.16)	0.45 (−0.63, 1.54)	0.413	
	Month 12	2.11 (1.26, 2.97)	0.17 (−0.68, 1.02)	1.94 (0.84, 3.04)	**0**.**001**	
6MWT distance in m	Month 6	450 (432, 469)	446 (428, 464)	4.27 (−8.03, 16.6)	0.496	—
MMAS-8 [0–8]	Month 3	0.11 (−0.08, 0.30)	0.05 (−0.13, 0.24)	0.06 (−0.19, 0.30)	0.657	0.329
	Month 6	0.14 (−0.05, 0.33)	0.25 (0.06, 0.44)	−0.10 (−0.35, 0.14)	0.410	
	Month 12	0.24 (0.05, 0.43)	0.09 (−0.10, 0.28)	0.15 (−0.10, 0.40)	0.231	
DASS-21 [0–126]	Month 3	1.94 (−0.85, 4.74)	2.64 (−0.14, 5.41)	−0.69 (−4.25, 2.87)	0.703	0.570
	Month 6	0.90 (−1.90, 3.69)	0.20 (−2.61, 3.01)	0.70 (−2.90, 4.31)	0.702	
	Month 12	0.02 (−2.82, 2.86)	1.87 (−0.96, 4.71)	−1.85 (−5.51, 1.81)	0.320	
Ejection fraction (%) in HFrEF at baseline**^c^**	Month 6	3.15 (−0.12, 6.43)	5.45 (2.01, 8.89)	−2.30 (−7.05, 2.46)	0.338	—
Left atrium size in cm in HFpEF at baseline**^c^**	Month 6	0.00 (−0.20, 0.20)	0.08 (−0.07, 0.30)	−0.11 (−0.38, 0.16)	0.416	—
Exploratory endpoints^b^						
Triglycerides in mmol/L	Month 6	−0.07 (−0.26, 0.13)	0.13 (−0.07, 0.32)	−0.19 (−0.44, 0.05)	0.121	0.096
	Month 12	−0.14 (−0.34, 0.05)	0.17 (−0.03, 0.37)	−0.32 (−0.56, −0.07)	**0**.**012**	
Fasting glucose in mmol/L	Month 6	−0.64 (−1.01, −0.26)	0.01 (−0.38, 0.39)	−0.64 (−1.12, −0.15)	**0**.**010**	**0**.**004**
	Month 12	−0.43 (−0.81, −0.04)	−0.10 (−0.49, 0.28)	−0.33 (−0.81, 0.16)	0.192	
HbA1c in mmol/mol	Month 6	−2.10 (−3.42, −0.79)	−1.18 (−2.51, 0.15)	−0.92 (−2.63, 0.79)	0.289	**0**.**044**
	Month 12	−4.35 (−5.68, −3.02)	−2.20 (−3.53, −0.87)	−2.15 (−3.87, −0.43)	**0**.**014**	
HDL cholesterol in mmol/L	Month 6	−0.02 (−0.08, 0.04)	−0.04 (−0.10, 0.02)	0.02 (−0.06, 0.09)	0.700	0.297
	Month 12	0.04 (−0.02, 0.10)	−0.02 (−0.08, 0.05)	0.05 (−0.02, 0.13)	0.183	
Left-arm systolic blood pressure in mmHg	Month 6	−0.89 (−4.52, 2.74)	1.71 (−1.95, 5.38)	−2.60 (−7.29, 2.08)	0.275	0.260
	Month 12	−2.73 (−6.41, 0.95)	−4.01 (−7.70, −0.32)	1.28 (−3.44, 6.01)	0.594	
Waist circumference in cm	Month 6	−2.26 (−3.67, −0.85)	−1.15 (−2.60, 0.30)	−1.11 (−2.92, 0.70)	0.230	0.429
	Month 12	−0.48 (−1.92, 0.96)	−0.01 (−1.47, 1.44)	−0.47 (−2.29, 1.36)	0.616	
Triglyceride/HDL cholesterol ratio	Month 6	−0.13 (−0.23, −0.04)	0.00 (−0.10, 0.09)	−0.13 (−0.25, −0.01)	**0**.**041**	**0**.**018**
	Month 12	−0.18 (−0.28, −0.08)	−0.02 (−0.12, 0.07)	−0.16 (−0.28, −0.03)	**0**.**014**	
Triglyceride–glucose index	Month 6	−0.08 (−0.13, −0.03)	0.02 (−0.04, 0.07)	−0.09 (−0.16, −0.03)	**0**.**006**	**0**.**009**
	Month 12	−0.07 (−0.12, −0.02)	0.01 (−0.04, 0.07)	−0.09 (−0.15, −0.02)	**0**.**012**	
EQ-5D-5L [0–100]	Month 6	0.04 (−2.70, 2.77)	−1.30 (−4.12, 1.52)	1.34 (−2.13, 4.80)	0.448	0.486
	Month 12	−1.60 (−4.36, 1.18)	−3.56 (−6.40, −0.72)	1.96 (−1.54, 5.47)	0.271	
EQ-VAS [0–100]	Month 6	−4.32 (−8.61, −0.04)	−1.31 (−5.65, 3.03)	−3.01 (−8.50, 2.48)	0.281	0.343
	Month 12	−2.46 (−6.83, 1.90)	−3.23 (−7.62, 1.15)	0.77 (−4.81, 6.35)	0.786	

^a^ *P*-values below 0.05 are presented in bold.

^b^Endpoints with at least two post-baseline measurements were analysed using linear mixed models, while those with only one post-baseline measurement were analysed using ANCOVA. Exceptions were the 6MWT distance, which was analysed using robust ANCOVA, and the Triglyceride/HDL cholesterol ratio, which was analysed using a robust linear mixed model, both due to the presence of extreme outliers. Endpoints were adjusted for baseline to correct for pre-existing baseline differences and additionally for covariates: age, sex, NYHA class, and education.

^c^These secondary endpoints were analysed within their respective subgroups and adjusted only for baseline values. Due to the small subgroup sizes, additional covariates were not included, unlike in the analyses of the whole study population.

All differences and changes are presented as absolute values in the original outcome units. The numbers in square brackets next to a variable name represent the score range. If not stated otherwise, endpoints were adjusted for baseline, age, sex, NYHA class, and education.

CI, confidence interval; DASS-21, 21-item Depression, Anxiety and Stress Scale; EHFScB9, nine-item European Heart Failure Self-care Behaviour scale; EQ-VAS, EQ visual analogue scale; EQ-5D-5L, the 5-level EQ-5D version; HbA1c, haemoglobin A1c; HDL, high-density lipoprotein; HFpEF, heart failure with preserved ejection fraction; HFrEF, heart failure with reduced ejection fraction; mITT, modified intention-to-treat; KCCQ-12, Kansas City Cardiomyopathy Questionnaire-12; MMAS-8, eight-item Morisky Medication Adherence Scale; 6MWT, six-minute walking test.

Subgroup analyses examined the difference in KCCQ-12 scores between participants in the intervention and control groups when stratified for the NYHA functional class. A significant difference (*P* = 0.023) was detected for KCCQ-12 scores between baseline and the 12-month endpoint for patients (11 in the intervention group vs. 11 in the control group) with NYHA functional class III (see Supplementary material online, *Table S1*), where the scores in the intervention group declined by only −0.19 points (95% CI: −9.94–9.56) but −16.60 points in the control group (95% CI: −27.73− −5.46).

### Self-care, disease-specific knowledge, and other self-reported outcomes

The secondary outcomes were among others, changes in scores on several questionnaires. Significant differences in self-care (based on EHFScB9 scores) were seen in the intervention group vs. the control group at both the 6-month (*P* < 0.001) and 12-month (*P* = 0.003) endpoints (*Table 3*). Also, for disease-specific knowledge, a significant difference was seen in the intervention group vs. the control group from baseline to the 12-month (*P* = 0.001) endpoint, but not from baseline to the 6-month point (*Table 3*). These results indicated that both self-care and disease-specific knowledge increased due to the intervention. No significant improvements were seen between groups in medication adherence (by MMAS-8), depression, anxiety, and stress (by DASS-21), or general QoL measure assessed as exploratory endpoint using EQ-5D-5L and overall health (by EQ-VAS) (*Table 3*).

### Functional cardiac measures, NT-proBNP, NYHA class, 6MWT, and metabolic syndrome

The secondary endpoints included changes from baseline to 6 months in EF in HFrEF patients, left atrium size in those with HFpEF, but no significant between-group differences were observed (*Table 3*). Changes in the number metabolic conditions and NT-proBNP from baseline to the 6- or 12-months endpoint were also assessed as secondary outcomes. The differences in mean NT-proBNP values at the 6- and 12-months endpoints were not significant between groups (*Table 4*). The probability of being in a lower NYHA functional class (class II-II vs. class III-IV) after 6 or 12 months in the programme did not differ between the groups (*Table 4*). Similarly, the change in 6-minute walk test (6MWT) distance from baseline to 6 months did not differ significantly between the groups (*Table 3*). In addition, changes in the number of metabolic syndrome conditions were not significantly different between the groups (*Table 4*).

**Table 4:** Estimates in secondary and exploratory endpoints for the mITT sample

Secondary endpoints						
Variable		Log-transformed estimated group mean after back-transformation (95% CI)		Difference, vs. control (95% CI)	*P*	Interaction *P*
		Intervention	Control			
NT-proBNP in pg/mL^a^	Month 6	441 (260, 747)	343 (202, 582)	97.7 (−50.6, 246.0)	0.195	0.270
	Month 12	476 (281, 807)	377 (222, 641)	98.9 (−62.6, 260.0)	0.228	

Variable		Incidence rate (95% CI)		Incidence rate ratio, vs. control (95% CI)	*P*	Interaction *P*
		Intervention	Control			
Metabolic syndrome conditions [0–5]^b^	Month 6	2.03 (1.70, 2.42)	2.19 (1.83, 2.62)	0.93 (0.76, 1.14)	0.466	0.800**^c^**
	Month 12	2.09 (1.75, 2.51)	2.33 (1.95, 2.78)	0.90 (0.73, 1.10)	0.313	

Variable		Probability of being in lower NYHA class (1 or 2 vs. 3 or 4) (95% CI)		Difference, vs. control (95% CI)	*P*	Interaction *P*
		Intervention	Control			
NYHA class^d^	Month 6	0.81 (0.70, 0.91)	0.83 (0.72, 0.95)	−0.03 (−0.12, 0.06)	0.562	0.478
	Month 12	0.77 (0.67, 0.87)	0.84 (0.73, 0.95)	−0.07 (−0.16, 0.02)	0.144	

Exploratory endpoints						
Variable		Estimated risk (95% CI)		Risk ratio, vs. control (95% CI)	*P*	Interaction *P*
		Intervention	Control			
EHFScB9 adequate score^e,f^	Month 6	0.36 (0.19, 0.68)	0.12 (0.04, 0.34)	3.08 (1.00, 9.43)	**0**.**049**	**0**.**006^c^**
	Month 12	0.62 (0.43, 0.90)	0.15 (0.06, 0.40)	4.13 (1.47, 11.59)	**0**.**007**	
Normal fasting glucose range^f,g^	Month 6	0.62 (0.38, 0.81)	0.30 (0.13, 0.54)	2.08 (0.98, 4.42)	0.057	0.063**^c^**
	Month 12	0.35 (0.16, 0.59)	0.17 (0.06, 0.40)	1.98 (0.73, 5.36)	0.180	

^a^NT-proBNP values were log-transformed due to strong right-skewness. All models were fitted on the log scale. Estimated marginal means were back-transformed (via exponentiation) to the original scale, representing geometric means. Group comparisons (contrasts) were performed on the log scale and then back-transformed to provide absolute differences between geometric means. Standard errors and confidence intervals were also back-transformed accordingly.

^b^Number of metabolic syndrome conditions was analysed using negative binomial regression model. Back-transformed group estimates represent incidence rates, and contrasts between groups are presented as incidence rate ratios (IRRs), with corresponding confidence intervals.

^c^The *P*-value for the interaction was obtained using a likelihood ratio test comparing a model with the interaction term to a nested model without the interaction.

^d^NYHA class was analysed using a cumulative link mixed model. We estimated the cumulative probability of being in NYHA class I or II (vs. III or IV). Group estimates reflect these cumulative probabilities, and contrasts represent differences in probabilities between the intervention and control groups.

^e^A score of ≥70.^25^

^f^The outcomes were analysed using a mixed-effects logistic regression model. Since the outcomes were common, ORs would overestimate the effect size. Therefore, we derived risk ratios (RRs) by re-gridding the marginal predictions to the response scale and computing contrasts on the log scale before back-transforming them to obtain RRs and corresponding confidence intervals, providing a more clinically interpretable measure of effect.

^g^Normal fasting blood glucose concentrations are between 70 mg/dL (3.9 mmol/L) and 100 mg/dL (5.6 mmol/L).^35^

*P*-values <0.05 are presented in bold.

CI, confidence interval; EHFScB9, nine-item European Heart Failure Self-care Behaviour scale; HDL, high-density lipoprotein; mITT, modified intention-to-treat; NT-proBNP, N-terminal pro-hormone of brain natriuretic peptide; NYHA, New York Heart Association functional classification.

### Hospital utilization

Hospital utilization (i.e. ER visits and hospitalizations due to cardiovascular causes) in the 12 months before the start and during the 12 months of the programme were compared (*Table 5*).

**Table 5:** Hospital utilization metrics, summed per group

Variable	Intervention	Control
	*n* = 86	*n* = 89
All ER visits, *n*		
Before the programme (12 months)	75	55
During the programme (12 months)	65	43
Unplanned ER visits, *n*		
Before the programme (12 months)	60	46
During the programme (12 months)	52	35
Hospitalizations, *n*		
Before the programme (12 months)	23	30
During the programme (12 months)	16	23
Hospitalization days, *n*		
Before the programme (12 months)	151	225
During the programme (12 months)	123	138

ER, emergency room; *n*, number.

The analysis of cumulative number of ER visits, hospital admissions, and the length of stay revealed no significant differences between the groups (*Table 6*).

**Table 6:** Summary and statistical analysis of hospital utilization during the digital health programme

Hospital utilization (6 months)				
Variable	Median (Q1–Q3; min, max)			
	Intervention, *n* = 86	Control, *n* = 89		
ER admissions	0 (0–0; 0, 2)	0 (0–0; 0, 1)		
Hospitalizations	0 (0–0; 0, 1)	0 (0–0; 0, 1)		
Length of stay in hospital (sum)	0 (0–0; 0, 9)	0 (0–0; 0, 7)		

Hospital utilization (12 months)				
Variable	Median (Q1–Q3; min, max)			
	Intervention, *n* = 86	Control, *n* = 89		
ER admissions	0 (0–0; 0, 3)	0 (0–0; 0, 1)		
Hospitalizations	0 (0–0; 0, 2)	0 (0–0; 0, 1)		
Length of stay in hospital (sum)	0 (0–0; 0, 17)	0 (0–0; 0, 13)		

Number of ER visits (12 months), analysed with a negative binomial model adjusted for the cumulative number over the 12 months prior to programme start, age, gender, NYHA, education, number of comorbidities, and number of reported medications				
Variable	Incidence rate (95% CI)		Incidence rate ratio, vs. control (95% CI)	*P*-value
	Intervention, *n* = 86	Control, *n* = 89		
All ER admissions	0.55 (0.36, 0.83)	0.44 (0.28, 0.69)	1.25 (0.73, 2.15)	0.416
Unplanned ER admissions	0.507 (0.32, 0.79)	0.36 (0.22, 0.58)	1.42 (0.79, 2.56)	0.246

Number of hospital admissions (12 months), analysed with a negative binomial model adjusted for: the cumulative number over the 12 months prior to programme start, age, gender, NYHA, education, number of comorbidities, number of reported medications				
Variable	Incidence rate (95% CI)		Incidence rate ratio, vs. control (95% CI)	*P*-value
	Intervention, *n* = 86	Control, *n* = 89		
All hospitalizations	0.20 (0.10, 0.40)	0.13 (0.06, 0.29)	1.49 (0.70, 3.18)	0.300

Length of stay in hospital (12 months), analysed with negative binomial model adjusted for: the cumulative number over the 12 months prior to programme start, age, and gender only due to convergence issues				
Variable	Incidence rate (95% CI)		Incidence rate ratio, vs. control (95% CI)	*P*-value
	Intervention, *n* = 86	Control, *n* = 89		
Length of stay	0.91 (0.36, 2.29)	1.02 (0.41, 2.57)	0.88 (0.25, 3.07)	0.844

CI, confidence interval; ER, emergency room; NYHA, New York Heart Association functional classification; Q1, first quartile; Q3, third quartile; *n*, number.

### Exploratory analysis: metabolic parameters and adequate self-care

A significant decrease in the intervention group vs. the control was found in the change of the triglycerides’ concentration at 12 months (*P* = 0.012), in HbA1c concentration at 12 months (*P* = 0.014), and for fasting glucose at 6 months (*P* = 0.010). The triglyceride-HDL ratio was decreased in the intervention group at 6 months compared with the control group by 0.13 (*P* = 0.041) and at 12 months by 0.16 (*P* = 0.014). Likewise, the triglyceride–glucose index was lowered in the intervention group by 0.09 compared with the control group both at 6 months (*P* = 0.006) and 12 months (*P* = 0.012) (*Table 3*).

Additionally, changes in the estimated risk of having an adequate EHF-ScB9 score or a normal fasting glucose range from baseline to the 6- or 12-months endpoints were analysed as exploratory endpoints. The RR for having an adequate EHF-ScB9 score was significant between the intervention vs. the control group, at both the 6-months endpoint (*P* = 0.049) with an RR of 3.08 (95% CI: 1.00–9.43), and at the 12-months endpoint (*P* = 0.007) with an RR of 4.13 (95% CI: 1.47–11.59). RRs in a normal fasting glucose range were not significant at either time point (*Table 4*).

### Safety

Approximately 80% of patients in both the intervention and control groups experienced at least one adverse event (AE) (see Supplementary material online, *Table S2*). The majority of AEs were mild or moderate in both groups, with severe AEs accounting for 13.4% in the intervention group and 18.9% in the control group. The most common AE was COVID-19 (18.3%), followed by infections (8.1%), atrial fibrillation (4.5%), HF complications (4.2%), chest pain (3.3%), and pneumonia (2.9%). The main serious adverse events (SAEs) were also due to HF complications (10.8%), atrial fibrillation (6.2%), and COVID-19 (6.2%). No AEs determined to be a result of the digital intervention were reported. SAEs were observed in 27.9% of patients in the intervention group and 25.8% in the control group.

## Discussion

This RCT was performed to determine whether a 6-months intervention period with the digital health programme, followed by a 6-months maintenance period, improved the QoL and clinical outcomes of HF patients. The digital intervention was very well received, as indicated by the high retention rates and compliance, suggesting that this approach has real potential to relieve the burden on outpatient HF clinics. While the programme did not improve health-related QoL as measured by the primary endpoint KCCQ-12, except in NYHA functional class III patients, improvements were seen in secondary endpoints such as self-care, disease-specific knowledge, and some key metabolic parameters (exploratory endpoints), such as triglycerides, fasting glucose, and HbA1c, when compared with SoC alone.

The improvement in self-care seen in the intervention group is a salient observation. Given the progressive nature of HF and the need for comprehensive disease management, as well as the increasing burden of disease due to the ageing population, an active role for patients in disease management is an ever-more important component of good clinical care.^38^ Another study found that a remote monitoring system aimed at improving self-care behaviour significantly improved self-care, health-related QoL, and also reduced the length of HF-related hospitalizations over time.^9^ Self-care has become an increasingly important metric in HF and includes adherence to medications, diet, self-management of symptoms, and exercise in addition to symptom monitoring and seeking assistance early when symptoms do occur.^39^

It is additionally of importance to evaluate patient acceptability of a digital approach, not least in elderly individuals, who make up a large proportion of HF cohorts. Therefore, the high in-app retention rates and compliance with remote monitoring are promising indicators that this approach was well received and this bodes well for the future development of digital therapeutics for this patient group.

The digital health programme also consisted of lifestyle education and coaching. This part was aimed at improving symptoms, well-being, and cardiovascular risk factors. Three key indicators of metabolic health, namely triglycerides, fasting glucose, and HbA1c improved with the use of the programme. The triglyceride–glucose index is strongly associated with CVD, including HF, prognosis, and mortality.^40^ For instance, in a study that included data from 11 937 adults in the United States, congestive HF was found to be associated with the triglyceride–glucose index (OR 1.90, 95% CI 1.18–3.04).^41^ The triglyceride–glucose index was significantly improved in our study, as was another indicator of metabolic health, the triglyceride-HDL cholesterol index. This further underscores the usefulness of the digital intervention in augmenting the increasingly important metric of metabolic health.

The results of this study suggest that digital health programmes have a future potential to relieve some of the burden on outpatient HF care in already overloaded healthcare systems, even though a clinical benefit was not demonstrated in specific HF characteristics, such as the 6MWT, and NT-proBNP levels and QoL, in this study. The feasibility study we previously performed with the same digital health programme suggested an effect on key HF symptoms (shortness of breath, fatigue, and leg oedema) in the KCCQ-12 over an 8-week period.^24^ The lack of direct clinical benefit in this study may, at least in part, be due to the fact that the large majority of patients in the trial had NYHA stages I-II and were already very compliant on good stable medications. Thus, improving clinical measures with a lifestyle intervention in a relatively short time was perhaps not realistic in this current cohort.

Interestingly, when we analysed health-related QoL in NYHA functional class subgroups *post hoc*, the intervention group exhibited a significantly smaller decrease in KCCQ-12 compared with the control group in those with NYHA class III, indicating that the digital intervention benefitted this subgroup, however this *post hoc* observation finding, while noteworthy, should be interpreted with caution. A trial recruiting more patients with NYHA stages III-IV might demonstrate a larger effect in a digital intervention group.

There is an increased need to improve outcomes in HF as the growing number of HF patients globally leads to high costs and a heavy burden on healthcare. New technologies, like the digital health programme such as we employed, can improve support of HF patients with symptom monitoring and necessary lifestyle changes, to complement standard medical care for outpatients with HF. Digital health interventions for HF patients are relatively new, and their effectiveness has not yet been extensively studied in large or longitudinal trials.^24^ A systematic review of studies analysing the impact of telemonitoring vs. SoC on self-care in HF patients found a positive effect on self-care although longitudinal studies were still lacking.^42^

Ultimately, improving prognosis and QoL in HF patients will likely be a combination of several measures, including medications, devices, education and lifestyle support with digital solutions that enhance self-care and allow exacerbations to be detected early. This underscores the importance of further developing and studying digital solutions such as this app in a large diverse population of HF patients. Thus, digital care will likely be just one part of a multi-modal effort to support the growing number of individuals with HF. Whether this will be with an app, or another medium is unclear but what is clear is that we now possess technology for significantly accentuated relationships with outpatients. What remains to be clarified is the best way to use this technology and which patients are the best fit.

Sidekick Health has developed digital health programmes for several other chronic diseases that have similarities to the digital health programme for HF, as they also include symptom monitoring and empower positive lifestyle changes. Initial results with these programmes have been promising as they resulted in an improvement of QoL and reduction of symptoms and severity in patients with atopic dermatitis,^43^ a reduction of stress and improvement of energy levels in patients with inflammatory bowel disease, an improvement of QoL and a reduction of stress and improvement of energy levels in patients with rheumatoid arthritis,^44^ an improvement in liver-specific and cardiometabolic health in patients with non-alcoholic fatty liver disease,^45^ and weight loss in overweight and individuals with obesity.^46^

This study has several limitations. First, the active intervention period was only 6 months, while in many of the placebo-controlled HF treatment trials including medications and cardiac resynchronization therapy, the separation of the treatment arms was only modest at around 6 months.^47–49^ A longer duration may, therefore, be needed to detect clinical benefits. Second, this was a rather well-treated and stable cohort with a relatively high KCCQ score at baseline and almost 90% of the participants were NYHA class I-II at enrolment. It may, therefore, have been rather optimistic to expect further improvement with lifestyle support alone in an intervention period of only 6 months. Third, the power calculation of the cohort size in this trial was based on a small feasibility study.^24^ These numbers may have fallen short of the numbers that are required to detect a difference in a RCT, particularly for hard endpoints such as cause-specific mortality. This study was conducted at Iceland’s only specialized HF outpatient clinic, limiting the cohort size. While a clear limitation, it also offered a unique setting to study this digital HF programme. The population was generally well-treated and stable (mainly NYHA class I–II), likely contributing to a ceiling effect and reducing the ability to detect changes in the KCCQ-12 score.

In conclusion, we found that the programme did not improve health-related QoL, except in exploratory analysis for those with NYHA class III. However, the programme was well accepted and did improve other important outcomes such as self-care, disease-specific knowledge, and key metabolic parameters. These are promising findings and support further efforts to both investigate and to begin integrating digital therapeutic tools into clinical practice or outpatients with HF. Larger clinical trials with a longer duration of the intervention period and more NYHA III-IV patients need to be conducted to assess the full clinical benefits in HF patients.

## Supplementary Material

ztaf063_Supplementary_Data

## Data Availability

The data will be shared upon reasonable request to the corresponding author.

## Lead author biography

Dr. David O. Arnar is the Chief of Cardiology at Landspitali—The National University Hospital in Iceland—and a Professor at the University of Iceland Faculty of Medicine. He completed his MD from the University of Iceland. He also holds a PhD from the University of Iceland and an Executive Master of Public Health degree from the University of Reykjavik. He completed his postgraduate training in internal medicine, cardiology, and cardiac electrophysiology at the University of Iowa Hospitals and Clinics in Iowa City in the United States.
