# Supplementary material for: Effect of a digital health intervention on outpatients with heart failure: a randomized, controlled trial
Source: Eur Heart J Digit Health. 2025 Jun 10;6(4):749–62. doi: 10.1093/ehjdh/ztaf063 (PMC12282350; doi:10.1093/ehjdh/ztaf063)
Supplement: ztaf063_Supplementary_Data [file ztaf063_supplementary_data.pdf]

Supplemental material

PP analysis results

PP analyses included 65 participants in the intervention group and 83 in the control group (**Figure 2**) to compare with the results of the mITT analyses. In the PP analysis, significant differences between the intervention and control groups were found for the change in triglycerides from both baseline to 6 months (change: -0.31, 95% CI: -0.55 to -0.06; p = 0.013) and from baseline to 12 months (change: -0.37, 95% CI: -0.62 to -0.13; p = 0.003). In the mITT analysis, a significant difference was only found for the baseline to 12-month endpoint.

PP analyses, like mITT analyses, detected significant differences between the intervention and control groups for the fasting glucose significant at 6 months (change -0.54, 95% CI: -1.05 to -0.04; p = 0.035), and HbA1c at 12 months (change -1.90, 95% CI: -3.73 to -0.06; p = 0.043). Similar to the mITT analyses, the self-care scores on the EHFScB9 scale were also significantly different in the PP analyses for both the change in mean score (at 6 months: 8.98, 95% CI: 4.37–13.60; p < 0.001, and at 12 months: 6.62, 95% CI: 1.89–11.30; p = 0.006) and the RRs of having an adequate EHFScB9 score (RR at 6 months: 3.36, 95% CI: 1.04–10.90; p = 0.043, and at 12 months: 4.21, 95% CI: 1.41–12.60, p = 0.010). Also, the scores on the HF disease-specific knowledge questionnaire were significantly different at 12 months in the PP analysis (difference: 2.22, 95% CI: 1.08–3.36; p < 0.001).

Supplemental Table S1. Subgroup analysis of between-group differences in KCCQ-12 scores across NYHA classes

	**Difference in KCCQ-12, vs control (95% CI)**	**P-value**	**Interaction p-value**
			0.085
NYHA class 1 (overall)	-4.96 (-11.54, 1.61)	0.138	
Month 3	-5.13 (-13.05, 2.78)	0.203	
Month 6	-5.16 (-13.31, 3.00)	0.214	
Month 12	-4.94 (-13.29, 3.42)	0.246	
NYHA class 2 (overall)	-0.22 (-5.54, 5.10)	0.936	
Month 3	2.43 (-4.07, 8.93)	0.463	
Month 6	-0.40 (-6.92, 6.12)	0.904	
Month 12	-2.89 (-9.48, 3.70)	0.389	
NYHA class 3 (overall)	5.32 (-5.84, 16.48)	0.348	
Month 3	-0.46 (-13.97, 13.05)	0.947	
Month 6	1.35 (-12.55, 15.25)	0.849	
Month 12	16.40 (2.31, 30.50)	**0.023**	

P-values below 0.05 are presented in bold.

CI, confidence interval; KCCQ-12, Kansas City Cardiomyopathy Questionnaire-12; NYHA, New York Heart Association.

Supplemental Table S2. Summary of adverse events per group

Variable	Intervention<br>n = 86	Control<br>n = 89
Patients with any AE, n (%)	69 (80.2)	69 (77.5)
Patients with SAE, n (%)	24 (27.9)	23 (25.8)
Total number of any AEs, n	186	148
Mild, n (%)	102 (54.8)	89 (60.1)
Moderate, n (%)	59 (31.7)	31 (20.9)
Severe, n (%)	25 (13.4)	28 (18.9)
Treatment-related, n	0	0
Total number of SAEs, n	33	32
Mild, n (%)	0 (0)	1 (3.13)
Moderate, n (%)	9 (27.3)	5 (15.6)
Severe, n (%)	24 (72.7)	26 (81.3)
Treatment-related, n	0	0

AE, adverse event; SAE, serious adverse event
